# An Agent-Based Model of Private Woodland Owner Management Behavior Using Social Interactions, Information Flow, and Peer-To-Peer Networks

**DOI:** 10.1371/journal.pone.0142453

**Published:** 2015-11-12

**Authors:** Emily Silver Huff, Jessica E. Leahy, David Hiebeler, Aaron R. Weiskittel, Caroline L. Noblet

**Affiliations:** 1 USDA Forest Service Northern Research Station, Amherst, Massachusetts, United States of America; 2 School of Forest Resources, University of Maine, Orono, Maine, United States of America; 3 Department of Mathematics & Statistics, University of Maine, Orono, Maine, United States of America; 4 School of Economics, University of Maine, Orono, Maine, United States of America; Université Toulouse 1 Capitole, FRANCE

## Abstract

Privately owned woodlands are an important source of timber and ecosystem services in North America and worldwide. Impacts of management on these ecosystems and timber supply from these woodlands are difficult to estimate because complex behavioral theory informs the owner’s management decisions. The decision-making environment consists of exogenous market factors, internal cognitive processes, and social interactions with fellow landowners, foresters, and other rural community members. This study seeks to understand how social interactions, information flow, and peer-to-peer networks influence timber harvesting behavior using an agent-based model. This theoretical model includes forested polygons in various states of ‘harvest readiness’ and three types of agents: forest landowners, foresters, and peer leaders (individuals trained in conservation who use peer-to-peer networking). Agent rules, interactions, and characteristics were parameterized with values from existing literature and an empirical survey of forest landowner attitudes, intentions, and demographics. The model demonstrates that as trust in foresters and peer leaders increases, the percentage of the forest that is harvested sustainably increases. Furthermore, peer leaders can serve to increase landowner trust in foresters. Model output and equations will inform forest policy and extension/outreach efforts. The model also serves as an important testing ground for new theories of landowner decision making and behavior.

## Introduction

Natural resources provide countless benefits to the public, but are often owned and managed privately. In the case of terrestrial ecosystems, these private landowners can potentially alter these resources in ways that will impact both the system itself and those that may depend on it for clean air, fuel, recreation, or water. Of total United States forestland, for example, non-corporate private woodland owners (PWOs) own 36% or 105 million ha with most of these woodland owners concentrated in the eastern United States [1]. PWOs may engage in forest management activities for a variety of reasons. These objectives include maintaining privacy, investing for the future, protecting the land from development, and harvesting timber for commercial use. Their management decisions impact the overall health and ecology of forested ecosystems. The harvesting decision, in particular, impacts the total available timber supply to markets such as bioenergy, pulp and paper, or sawlog production [2] and forest health and structure. Some PWOs are heavily influenced by information around them while others make decisions about their land without hiring a forest professional. Acceptance of information and thereby influence is potentially a function of trust in the natural resource professional [3], perceived self-efficacy regarding the activity or behavior [4], and the actual outcome of the interaction.

During this decision making process, landowners can be influenced by professionals, neighboring landowners, or other influential peer leaders [5]. These landowner communication channels and social network structures are just now being explored in the literature. The results of many studies demonstrate where landowners prefer to receive information, how they use the information they receive, who they interact with, and how much they trust information and individuals with whom they interact [6, 7]. Of particular importance is the role of influential acquaintances or peer leaders in communities [8]. Recent work suggests that boundary spanners are also increasingly important for providing information to landowners and connecting landowners with resources for land management decisions and actions [9]. Boundary spanners are individuals that create new connections between organizations and individuals [10]. Boundary spanners or peer leaders could help landowners make better informed management decisions.

### Background

PWOs are decision-makers whose decisions impact system-wide behavior; the behavior of the system emerges from the decisions made by individual landowners. PWOs are also autonomous (having the freedom to make independent decisions from one another) and heterogeneous (having different demographic characteristics). They also have bounded rationality; they do not often have full information to make harvesting decisions. These aspects make PWO management decisions an ideal research scenario for agent-based models (ABMs) [11] (Table 1). There are many factors, both endogenous and exogenous, that influence forest management. Consequently, ABMs are an excellent method to explore and understand the complexities of these interacting factors. Additionally, social interactions and peer-to-peer networks can be sensibly modeled in an ABM context. In ABMs, agents can interact and follow their individual decision rules, allowing the model user to see the outcomes of both individual decisions and agent-agent interactions.

**Table 1:** Dimensions of agent-based modeling.

Dimension	Applicability
Heterogeneity of agents	Forest landowners have heterogeneous demographics and as a result, will respond differently to contact with foresters, peer leaders, and neighbors.
Autonomy of agents	Forest landowners make independent decisions about timber harvesting, although they are influenced by other agents
Explicit space	Forest landowners are influenced by their adjacent neighbors to harvest, not to harvest, and to update their sustainability values and trust in natural resource professionals and peer leaders
Local interactions	Forest landowners interact with adjacent neighbors to share information.
Bounded rationality of agents	Forest landowners can only decide to harvest based on the information they are given in the model.

The application of ABM to forest management behavior is relatively new. Satake et al. [12] studied interaction between neighboring parcels on patterns of harvesting and found that the harvest rate was higher in a weakly-connected society. Mayer and Rouleau [13] found that information flow between landowners changes landscape-level forest structure, given certain sociological parameters. There is also a growing literature that explores wood markets using ABMs [14]. Finally, Leahy et al. [15] modeled timber harvesting behavior in Maine and demonstrated potential impacts of forest pests and economic disturbances on harvesting levels of PWOs. These studies demonstrate the potential for ABMs to incorporate PWO demographics and social network concepts to better understand the ramifications of individual decision-making on a forested landscape. However, none of these models look specifically at the various social networks and communication channels of landowners to assess how knowledge-transfer interactions influence forest management behavior.

### Study Objectives

This study aims to build a model of PWO decision-making, particularly measuring and experimentally manipulating landowner information sources, trust in information sources, and influence of information on decision-making. The objectives of this study were to: 1) build an ABM of PWO forest management decisions incorporating interactions with natural resource professionals, peer leaders, and community members; 2) perform verification testing and sensitivity analysis on the model; 3) evaluate how the number of foresters and peer leaders in the model influence outcomes; and 4) demonstrate model capacity for future experimentation. To achieve these objectives, we will 1) provide a brief overview of modeling approaches for natural resource management with particular focus on timber harvesting (see **Modeling approach** Section); 2) describe the ABM structure, core assumptions, and general behavior (see **Model ODD** Section); 2) verify model behavior and complete a sensitivity analysis (see **Model verification and initial experimentation** Section); and 3) demonstrate the usefulness of the model (see **Model validation** Section). This model will provide an important testing environment for understanding how social interactions and decision-making processes affect the management of natural resources. This model will also provide a flexible framework for adding empirically based network topologies and utility maximization functions.

### Modeling approach

Natural resource management studies often incorporate data across multiple scales from individual behavior to community or regional collaboration and governance. At the individual-level, computational modeling is increasingly important for understanding the linkages between behavior and natural resource availability and quality [16]. Empirical models of individual behavior, although still computational in nature, are helpful, but are static. In other words, they only provide a snapshot in time. Furthermore, they are often deterministic with no interaction between social and ecological factors [16].

In contrast, ABMs can provide an adaptive framework of rules based on empirical data to reflect our current understanding of behavioral mechanisms and relationships that lead to decisions, and allows the attribution of landscape-scale change to individual-level behavioral components. ABMs are built upon early computer science cellular automata models and object-oriented programming. ABMs can be empirical or theoretical in nature, but always involve computer simulation of individuals (agents) that interact based on prescribed rules [17]. This bottom-up approach was developed as an alternative to top-down empirical models of behavior in concert with increasing computing power and the emerging field of complexity science [18]. Typically, system-level or landscape-scale features emerge from the interactions of agents in the model. ABMs are a central tool to the study of coupled human and natural systems, and are increasingly used to study the impact of human behavior on natural resources [19].

In ecology, ABMs have been used since the 1980s to understand phenomena such as the flocking behavior of birds [20] and habitat selection of trout [21]. ABMs have also been used extensively to study social phenomena such as segregation [22] and marriage [23]. It is only recently that ABMs combine both natural and social processes to understand an overall system. Grimm & Railsback [17] outline the ideal properties of an ecological system or question that warrant an ABM approach (Table 1). An ABM approach clearly fits the attributes of private forest management that arise from the individual decisions of forest landowners and their interactions with natural resource professionals, peer leaders, and neighbors (Table 1).

### Modeling constructs

The timber harvesting behavior, a specific management decision with far-ranging impacts on forest resources at a landscape scale, of PWOs has a long history of research [24]. One approach is to look at the entire ‘decision making environment’ of PWOs [25]. Some have analyzed the economic literature on the decision to harvest [26, 27], concentrating on utility maximization and finding which landowner characteristics significantly predict harvesting intention. Despite confirmed statistical relationships between demographic and cognitive characteristics and the intention to harvest, the final harvesting decision or behavior is highly moderated by external factors such as interactions with peers, professionals, and local community members.

When making a timber harvesting decision, PWOs may receive and share information about their woodland with a variety of individuals including, but not limited to consulting foresters, state agency foresters, extension and outreach professionals, neighbors, friends, community members, family, and other non-forestry professionals such as tax specialists [8]. Furthermore, these landowners may also receive information from written sources such as websites, newsletters, e-mail listserves, and scientific literature [28]. Unsurprisingly, landowners seem to prefer face-to-face interactions for information [7], but judge interactions less by scientific credibility or professional expertise and more on social impressions [29]. Often, landowners prefer a combination of practical advice from peers and technical information from professionals [30].

The role of social networks in private woodland owner decision-making is typically to provide information or knowledge. Social network analysis (SNA) is a tool used to describe relationships between individuals and the significance or role of these relationships [31]. Research using SNA to understand forest landowner decisions provides numeric results about landowner relationships in a social network [8, 32, 33], useful in tailoring outreach information. For example, the role of social networks in timber harvesting suggests that active forest managers are an important conduit to more passive forest landowners [34]. Landowners report somewhere between zero and five influential people in their network regarding forest management decisions [34]. The numeric results of these SNAs are potentially useful in parameterizing ABMs of landowners’ decision-making. Although this model does not incorporate a specific network topology, SNA theory drives the choice to incorporate landowner interactions as a central feature of the model.

The outcome of interactions between PWOs and others are heterogeneous. Some landowners may have very positive interactions with a forester, while others will not. Trust is one potential mitigating factor in whether or not the information imparted by an individual will be absorbed and acted upon by another individual. In psychology, trust has been defined as “an expectancy held by an individual or group that word, promise, verbal, or written statement of another individual or group can be relied on” [35]. Nearly all scholars agree that trust is developed over time and influenced by personality traits or social experiences, and that trust is loosely defined as an expectation held by an individual. Expectation implies a perception of future conditions; in fact, trust is often linked to the uncontrollability and unpredictability of the future and thus can be defined as “a bet about the future contingent actions of others” [36].

Finally, Rousseau et al. [37] found a definition that worked across disciplines: “Trust is a psychological state comprising the intention to accept vulnerabilities based upon positive expectations of the intentions of behavior of another.” [37]. It is worth noting that distrust is not necessarily the opposite of trust. If distrust is assumed to be simply the opposite of trust [36], it implies that distrust can conceptually exist without trust. Conversely, it is easy to imagine a more practical definition of distrust as the ‘loss of trust’, which implies that trust must first exist and be defined [38]. This may seem a trivial distinction, but will undoubtedly affect results of a modeling effort if improperly defined and parameterized.

## Methods

First, the model description is provided, following the ODD (Overview, Design concepts, Details) protocol [11, 39]. Then, verification and sensitivity analysis procedures are described. This model was built using NetLogo software [40], Version 5.2. Model code is available via website repository, “Open ABM CoMSES Computational Model Library”, to interested users (https://www.openabm.org/model/4710/version/1/view). Empirical data for parameterizing landowner characteristics was analyzed from the Penobscot County Survey dataset in the state of Maine, U.S. [41].

### Modeling ODD

#### Purpose

The purpose of this model was to better understand forest management at a landscape-scale based on the individual decisions of PWOs. The landscape-scale pattern of timber harvesting was based on individuals’ decisions to harvest timber, which in turn is affected by interactions with professionals, peer-to-peer networks, and internal cognitive factors such as trust in information, and predisposition to sustainably manage timber.

#### Entities, state variables, and scales

There are three types of agents in this model. PWOs are represented by the actual forest patches on the landscape, one owner per patch. The PWO’s forest is also represented by the patches on the landscape. The second type of agent is a professional forester. This could be a certified forester, an extension forester, or other forestry professional. This agent type can move around the landscape and ‘visit’ landowner patches at which time an interaction and exchange of information occurs. The final agent type is a peer or community member of private forest landowners. These agents also move around the landscape and interact with private forest landowners giving them information with which to make a decision.

Each patch represents a forest parcel owned by an individual landowner (or family). These patches are assigned an acreage based on survey data [41]. The landscape typically has over 33 thousand ha (800,000 acres) of woodland represented. The only overall environmental (contextual) variable that influences the behavior and dynamics of the PWOs is the harvestability of a parcel. This parameter looks at forest age and type, and takes a value for whether or not the patch has enough tree growth for a harvest. The forest age updates at the end of each tick, which represents a year, as part of a regrowth submodel.

The overall collective in this model are called “Influencers.” This includes foresters and peer leaders. These agents influence landowners’ decision to harvest timber. The other landowners themselves share opinions that may impact trust, which in turn may impact the final decision to harvest timber.

#### Process overview and scheduling

This model represents a theoretical landscape of different-sized forest patches. Each step in the model represents 1 year and the model runs for 400 years or 4–5 harvesting rotations. Within a year, the following occurs in this order (See Submodels section, for more detail):

- Variables reset (Landowner age and parcel harvest status)
- Pre-Interaction harvest decision (logistic regression)
- Foresters Interact with Landowners
  - Move to a patch
  - Influence sustainability value
  - Influence harvest-decision
  - Influence the landowner’s trust in foresters
- Peer Leaders Interact with Landowners
  - Move to a patch
  - Influence sustainability value
  - Influence the landowner’s trust in peer leaders
  - Influence the landowner’s trust in foresters
- Community Interaction (positive, negative, or neutral information spread)
  - Share an opinion
  - Influence the landowner’s trust in peer leaders and foresters
- Final Harvest Decision
  - Assess harvestability and sustainability of the patch to make a final decision
- Variables are updated and stored

#### Design concepts

The basic principles in this model include the theory of planned behavior, the theory with the most empirical support for translating demographic variables into a behavioral intention to an actual decision or harvesting behavior [24]. Additionally, the concept of trust is used as a threshold over which the landowner will change their behavior due to the influence of another individual. For example, when a forester recommends a timber harvest to a landowner, the landowner will not accept this recommendation unless they trust the forester. This model also uses the basic principle of sustainable harvesting and ecological forestry [42]. Sustainable harvesting ensures that enough advanced regeneration of desirable species exist such that forest composition and structure can be maintained on a rotational basis. Finally, this model incorporates aspects of social network theory, particularly the concept that information is passed along networks and can be passed among individuals with weak ties (a community member) just as often and influentially as among individuals with strong ties (friends or family) [43].

Model outputs include harvest percentage (sustainable and unsustainable) and landowner trust in foresters and peer leaders. Harvest percentage is more tightly imposed by model rules and depends more on the initialization of demographic variables. Landowner trust is an emergent property of the model. Trust depends on number of forester/peer leader interactions with landowners, and a stochastic element governing how much trust increases or decreases as a result of this interaction. Landowner trust in foresters and peer leaders is measured on each tick. Trust is classified as high (trust > 0.5) or low (trust < 0.5) for both entities and is aggregated to a percentage of landowners with high/low trust at each time step.

Agents adapt their sustainability and trust values based on interactions with foresters and peer leaders. This, in turn, influences their decision of whether or not to harvest their forested parcel. If an agent harvests, the parcel is set back to a harvestability of 0 and forest age is, likewise, reset. The model currently does not have a measure of utility or economic gain for the landowner.

If agent trust in a forester or peer leader has remained above a probability threshold of 0.7 for 5 or more ticks, the agent’s trust is permanently set to 1.0. Similarly, if an agent has a trust level below 0.3 for 5 or more ticks, the agent’s trust is permanently set to 0. This represents a learning mechanism whereby trust or distrust moves into a belief rather than a more transient attitude or opinion. In this model, agents have an implicit prediction that trusting a natural resource professional will lead to a better harvesting outcome. Agents ‘know’ their demographic conditions and their resource availability. They ‘sense’ whether or not community members and certain neighbors have a positive or negative experience with foresters and peer leaders. Agents also ‘sense’ information from contact with a forester, such as the market price for wood and how to conduct their harvest in some cases leading to the decision to harvest.

Foresters and peer leaders can interact with patches (landowners) to share information (e.g. contact information of a logger) that in turn influences the likelihood that a landowner will harvest sustainably. Landowners interact with each other to spread information about foresters/peer leaders.

Stochasticity is used to create the initial conditions of the landscape, within defined ranges. Stochasticity is also used to move agents around the landscape, with a pre-determined number of visits to patches. Stochasticity is used to assign demographic characteristics to agents, but within defined proportions from empirical data. Stochasticity is also used to determine whether or not an agent changes a parameter using trust as the probabilistic threshold. For example, if a forester is on a given patch, the model will run an if-then rule: *If random-float 1 < Forester-trust*, *set sustainability 1*. This picks a random number between 0.0 and 1.0 and if that number is less than the agent’s trust in the forester, the agent will change its sustainability value to 1, meaning they will only harvest if their forest is ready for harvest. Therefore, the higher the trust value, the more likely they are to be influenced by the forester to harvest sustainably. However, there is still a chance that a landowner with 90% trust in foresters won’t change their sustainability value.

Variables are initialized based on empirical data when available, personal communication, and a pseudo-random number generator, bounded by known distributions or ranges (Table 2). This model uses data from the Penobscot County Survey of Maine landowners who own forestland in Penobscot county [41]. These data are used to initialize landowner demographic characteristics and parameterize the logistic regression equation. Forester and peer leader visits were initialized using data from personal communication with foresters (stand and public) and state forestry data in Maine.

**Table 2:** State variables of agents.

Agent	Variable, *Netlogo name*	Description	Source for Initialization
Landowner	Timber harvesting ownership objective *inct*	Sets whether or not timber harvesting is an important ownership objective (range 0–5, mean 2.66, Poisson distribution)	Survey Data
	*age*	The age of the landowner (mean = 56.96, sd = 12.68, normal distribution)	Survey Data
	How long the land has been in the family, *lngfam*	How long the land has been in the family (Mean = 31.46, Poisson distribution)	Survey Data
	Absentee landowner, *live*	Whether or not the landowner lives on the property (1 = live on the land, 2 = seasonal, 3 = absentee, 4 = other; Mean = 2.04).	Survey Data
	Parcel size, *ha*	Size of forest parcel (Mean = 35.50, sd = 85.49)	Survey Data
	*Harvestability*	If the parcel is ready for harvest, ready for pre-commercial harvesting, or not ready (0 = not ready, 1 = pre-commercial thinning, 2 = mature and ready)	Stochastic
	*Forest-age*	The age of the forested parcel (0–60 years)	Stochastic, up to 60
	*Forest-type*	The forest type (0 = hardwood dominated, 1 = softwood dominated)	Stochastic
	*Patch-harvested?*	A Boolean variable that takes on a TRUE if the patch is harvested on a tick and FALSE if not	N/A
	*pre-harvest*	The value generated by a logistic regression equation determining whether or not a landowner is inclined to harvest his/her parcel	Survey Data
	*Sustainability*	A value indicating whether or not a landowner is inclined to sustainably or unsustainably harvest their property (0 = unsustainable, 1 = sustainable)	Stochastic
	*Forester-trust*	The likelihood a landowner will trust information conveyed by a forester. Represented by a probability from 0–1. Randomly assigned at first, but then adapts after each time step.	Stochastic
	*Peer leader-trust*	The likelihood a landowner will trust information conveyed by a peer leader. Represented by a probability from 0–1. Randomly assigned at first, but then adapts after each time step.	Stochastic
	*Psychdist*	The psychological distance of each landowner with respect to timber harvesting. Represented by a probability from 0–1. Randomly assigned until data is available.	Stochastic
Forester	*F visits*	The number of landowners a forester can visit in one year (range 10–500, drawn randomly).	Personal Communication
Peer leader	*C visits*	The number of landowners a peer leader can visit in one year (range 1–5, drawn randomly).	Personal Communication

#### Submodels

1. Variable reset: Variables that hold information about whether or not a patch was harvested or harvested sustainably are set back to FALSE or 0. Age is advanced by 1 year. If age reaches 100 years old, the agent dies and the age is set back to a random value to represent transfer of the parcel to a new landowner. If a forest is harvested, forest age is reset to 0.

2. Pre-Interaction Harvest Decision: A logistic regression equation takes parcel size, age, ownership objectives, length of time land has been in the family, and whether or not the landowner lives on their land and produces a probability the landowner will decide to harvest timber:

H = −3.065 + 0.001(A) + −0.30(L) + 0.282(I) + 0.03(AG) + 0.01(F)

where A is the size of parcel owned, L is whether or not a landowner lives on the property (0–4 scale), I is the timber ownership objective (0–5 scale), AG is the landowner’s age, and F is how long land has been in the family. The model fit well and had an area under the curve of 0.75 (p < 0.001). A random number is then chosen between 0.5–1. If this number is less than the probability a landowner will harvest, the harvest decision is changed to true, otherwise it is false.

3. Foresters Interact: The number of forester-landowner interactions is based on state averages for Maine. Private licensed foresters work with 50–100 clients annually (K. Ellis, personal communication, 2015). State or district foresters work with 100–500 landowners annually, but there are only 10 district foresters in Maine compared to 794 licensed foresters (M. Moesswilde, personal communication, 2015). The model selects somewhere between 50 and 100 landowners to visit during initialization. Foresters land on a random patch and change three potential things about a landowner. First, the forester interaction will change the landowner’s sustainability value to 1 if their forester-trust value is higher than a random number. Second, the forester will change the landowner’s harvest decision towards harvesting (only when their forest is mature) if their forester-trust value is higher than a random number. Finally, trust in that forester will either increase or decrease based on a random Bernoulli value. The landowner could have a positive or negative experience with the forester and there is no empirical data to support the probability of negative vs. positive interactions. The amount that trust increases or decreases is a random number between 0–0.75, and 0–0.5 respectively, but cannot increase the trust probability above 1.0 or below 0 (Table 3). It is assumed that while gaining a landowner’s trust can occur in large increments due to the cognitive desire for confirming evidence once an opinion forms, losing a landowner’s trust occurs slowly until it is lost completely.

**Table 3:** Agent rules during landowner-forester and landowner-peer leader interaction.

Agent	Target	Rule	Outcome
Forester	Visits Landowner	If a randomly chosen number is < landowner’s forester-trust	Set sustainability to 1
Forester	Visits Landowner	If a randomly chosen number is < landowner’s forester-trust	Set harvest-decision to TRUE
Forester	Visits Landowner	If the probability of forester-trust is higher than a random number	Set forester-trust to its old value + a random increment between 0–0.75
Forester	Visits Landowner	If the probability of forester-trust is lower than a random number	Set forester-trust to its old value−a random increment between 0–0.5
Peer Leader	Visits Landowner	If a randomly chosen number is < landowner’s forester-trust	Set sustainability to 1
Peer Leader	Visits Landowner	If the probability of Peer Leader-trust is higher than a random number	Set Peer Leader-trust to its old value + a random increment between 0–1.0
Peer Leader	Visits Landowner	If the probability of Peer Leader-trust is lower than a random number	Set Peer Leader-trust to its old value−a random increment between 0–1.0
Peer Leader	Visits Landowner	If the probability of Peer Leader-trust is higher than a random number	Set forester-trust to its old value + a random increment between 0–1.0

**Fig 1:**
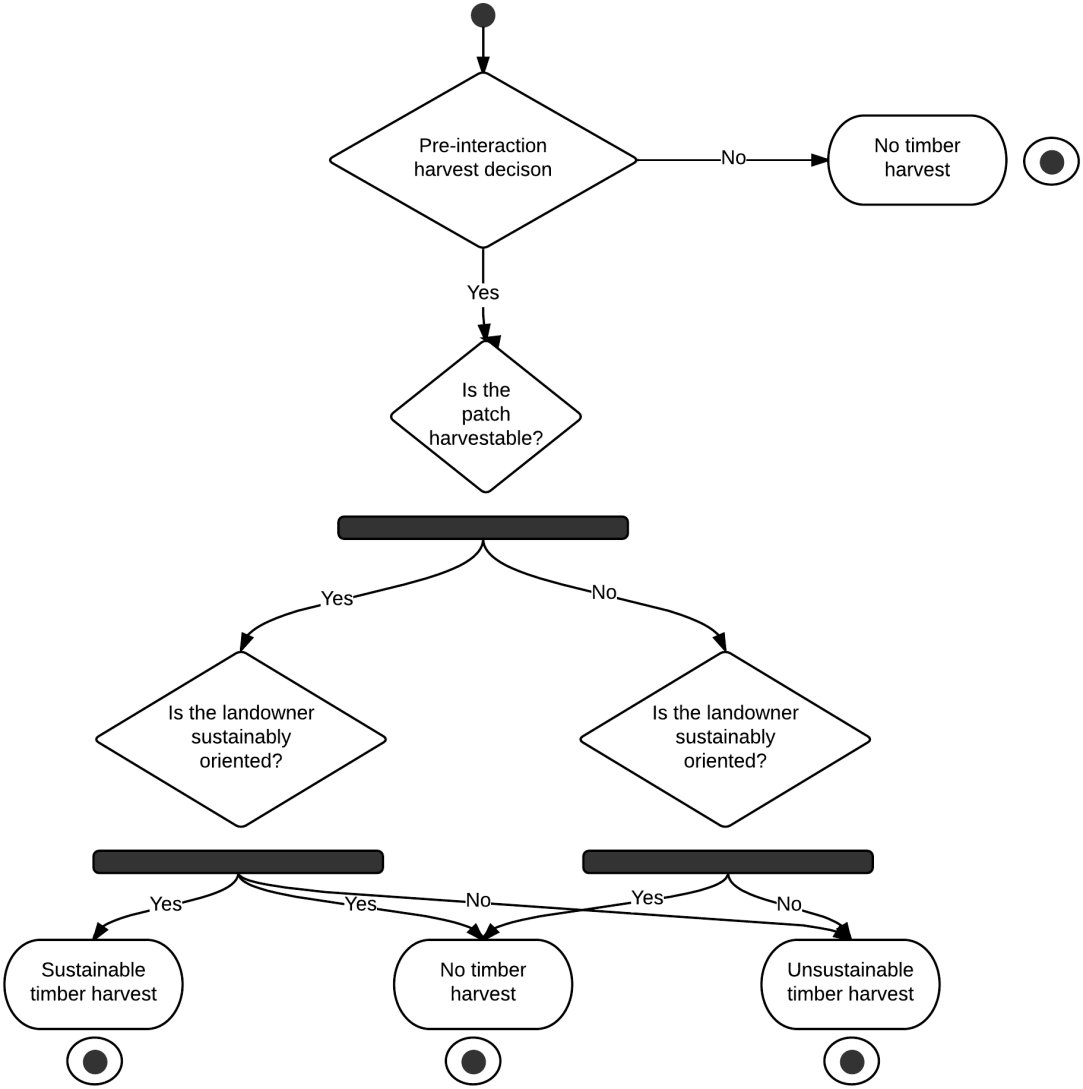
An activity diagram of the final harvest decision rules.

4. Peer Leader Interaction: The number of peer leader-landowner interactions is based on averages from the Keystone Peer leader program in Massachusetts (David Kittredge, personal communication, 2015), which surveyed peer leaders as to how many referrals they made in their conversations with community members about conservation options. These interactions are lower than forester interactions, set between 1 and 5 landowners per year. The peer leader potentially changes three values. First, the peer leader interaction will change the landowner’s sustainability value if the landowner’s peer leader-trust value is higher than a random number. Second, the trust in that peer leader will either increase or decrease based on a Bernoulli value. The landowner could have a positive or negative experience with the peer leader. Finally, the peer leader could change how much a landowner trusts a forester. Given that they are typically positive information spreaders, forester-trust will either increase or stay the same. The amount that trust increases or decreases is a random number between 0–1.0, but cannot increase the trust probability above 1.0.

5. Community Interaction: Based on existing NetLogo Rumor Mill code [40], landowner agents either share an opinion in a random interaction across the landscape or in an adjacent neighbor interaction (but not both). In this step, community members share an opinion. If a landowner is influenced by that opinion, they will update their Forester-trust and Peer leader-trust according to whether or not the opinion is positive, negative, or neutral. This does not change the harvest decision. The amount that trust increases or decreases is a random number between 0–1.0, but cannot increase the trust probability above 1.0. Originally, the model user could define whether or not the opinion would be spread globally or locally. A sensitivity analysis demonstrated little effect on model output, so this was changed to a stochastic operator.

6. Final Harvest Decision: The landowner updates the pre-interaction decision with two parameters. First, the harvestability of the patch is queried. This value tells the landowner whether or not there is enough tree growth on the patch to harvest sustainably. Second, the sustainability value of the landowner is queried. This value tells the landowner whether or not they would like to harvest timber when it is mature and ready for harvest or if they would like to harvest when there is not enough advance regeneration and they risk conversion to an undesirable species mix. The rule sets lead to an updated harvest decision (Fig 1). At the end of the harvest decision, each landowner assesses their trust value from the last 5 years and possibly converts trust/distrust to a permanent value.

### Verification, validation, and testing

Computational modeling of real-world systems typically involves a set of simplifying assumptions to render these systems into a computer-based application. These assumptions often alter model output and in some cases affect the generalizability of the model back to the real world. To defend a model’s generalizability, the process of documenting the validity of the model and verifying the models’ accuracy is critically important. This process, Validation, Verification, and Testing (VV&T), occurs throughout the modeling cycle [44]. The goal is to ensure predictive validity of the model or to consistently illustrate the differences between two models.

Verification includes testing the model to ensure that each sub-model performs in the way it was intended, performing sensitivity analyses, and assessing software or other processing errors. Model testing also involves changing model parameters to discover logical fallacies, and attempting to break the model to discover weaknesses or programming issues. Verification of model parameters was checked by sending code to other agent-based modeling researchers to check code, exporting values of each model variable during model runs, and performing sensitivity analyses. When model outputs (percent harvested and percent trust) met assumptions of normality, an Analysis of Variance (ANOVA) was performed on output of parameter sweeps to assess if different parameter levels led to statistically significantly different model output. Finally, a boosted regression tree [45] was used to determine the variables that contributed most to model output.

Validation is the process by which model assumptions and content are analyzed and documented to show, when possible, empirical relationships or cases where the model matches documented values in scientific research or from subject matter expertise. Construct validity is a check to see if the model appears to be a reasonable imitation of a real-world system. Content validity is a check to determine if the model accounts for all the theoretical constructs and operational definitions. Criterion, concurrent, and convergent validity are techniques determine if the model correlates with other similar or dissimilar models, if the model predicts what it claims to, and if the model is correlated with current empirical data.

## Results

### Model verification and initial experimentation

Model output on percent harvested (overall, sustainably harvested, and unsustainably harvested) was analyzed beginning with year 10, to account for initial harvesting of mature parcels not indicative of the overall trend. Parameter sweeps of key model variables (100 repetitions per level per variable) resulted in consistent model output ranges (Table 4).

**Table 4:** Aggregated model output from parameter sweeps of key model variables.

Variable	Levels	Percent of landowners that trust forester (min- max, mean, sd)	Percent of landscape harvested (min—max, mean, sd)	ANOVA results
*Forester Visits*	*25*, *50*, *100*	53.0–60.89, 56.7, 1.34	5.4–21.60, 12.72, 4.68	Trust: F = 2.211, p = 0.110 Harvest: F = 0.244, p = 0.783
*Peer Leader Visits*	*10*, *25*, *50*	53.0–60.89, 56.7, 1.34	5.40–21.60, 12.68, 4.68	Trust: F = 38.53, p < 0.001 Harvest: F = 0.229, p = 0.780
*Number of Foresters on the landscape*	*0*, *2*, *4*, *6*, *… 38*, *40*	51.89–63.53, 57.02, 1.92	4.28–28.39, 9.16, 4.24	Trust: F = 5942, p < 0.001 Harvest: F = 2260, p < 0.001
*Number of Peer Leaders on the landscape*	0–5	50.55–60.97, 56.08, 1.79	5.48–21.55, 12.85, 4.60	Trust: F = 7458, p < 0.001 Harvest: F = 6.36, p < 0.001
*Opinion-type*	Positive, Negative, Neutral	47.35–60.22, 53.39, 2.95	5.60–21.38, 12.94, 4.60	Trust: F = 37819, p < 0.001 Harvest: F = 6.03, p = 0.002

Number of landowners foresters and peer leaders visited in each tick were not statistically significantly different with regard to percent of the landscape harvested and trust in foresters, except number of landowners peer leaders visited was statistically significant with regard to landowner trust in foresters. Total number of peer leaders and foresters on the landscape were statistically significantly different for both percent harvested and landowner trust in foresters (Table 4), warranting further exploration.

**Fig 2:**
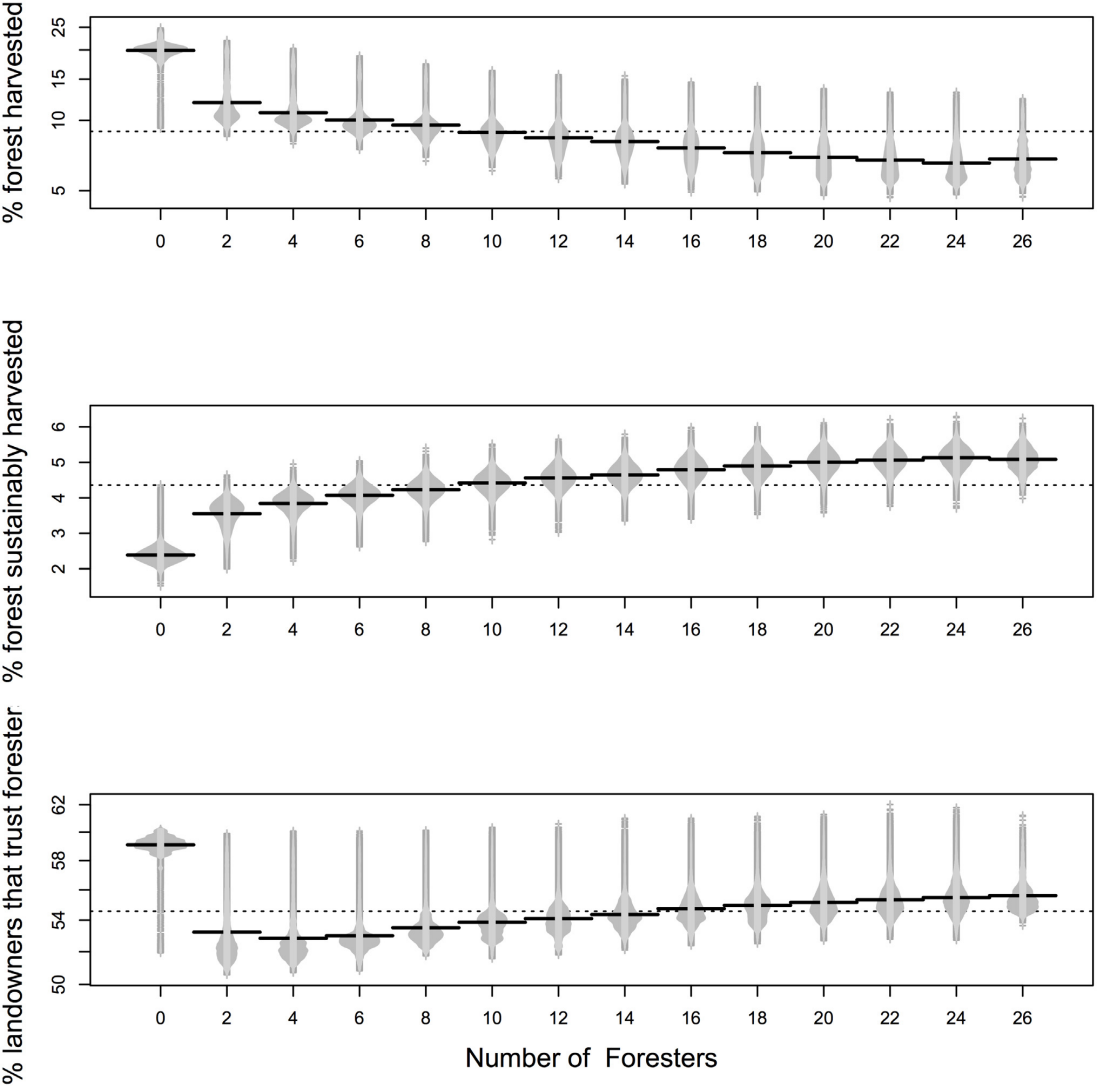
Beanplots [46] showing the relationship between number of foresters, harvesting, and trust. The top and middle graphs shows the relationship between number of foresters and percent of the forest harvested and harvested sustainably. The bottom graph shows the relationship between number of foresters and the percentage of landowners that trust foresters. All other parameters are held constant and averaged for the last time step over 100 simulations per forester level. The dashed line is the overall mean of all forester levels.

**Fig 3:**
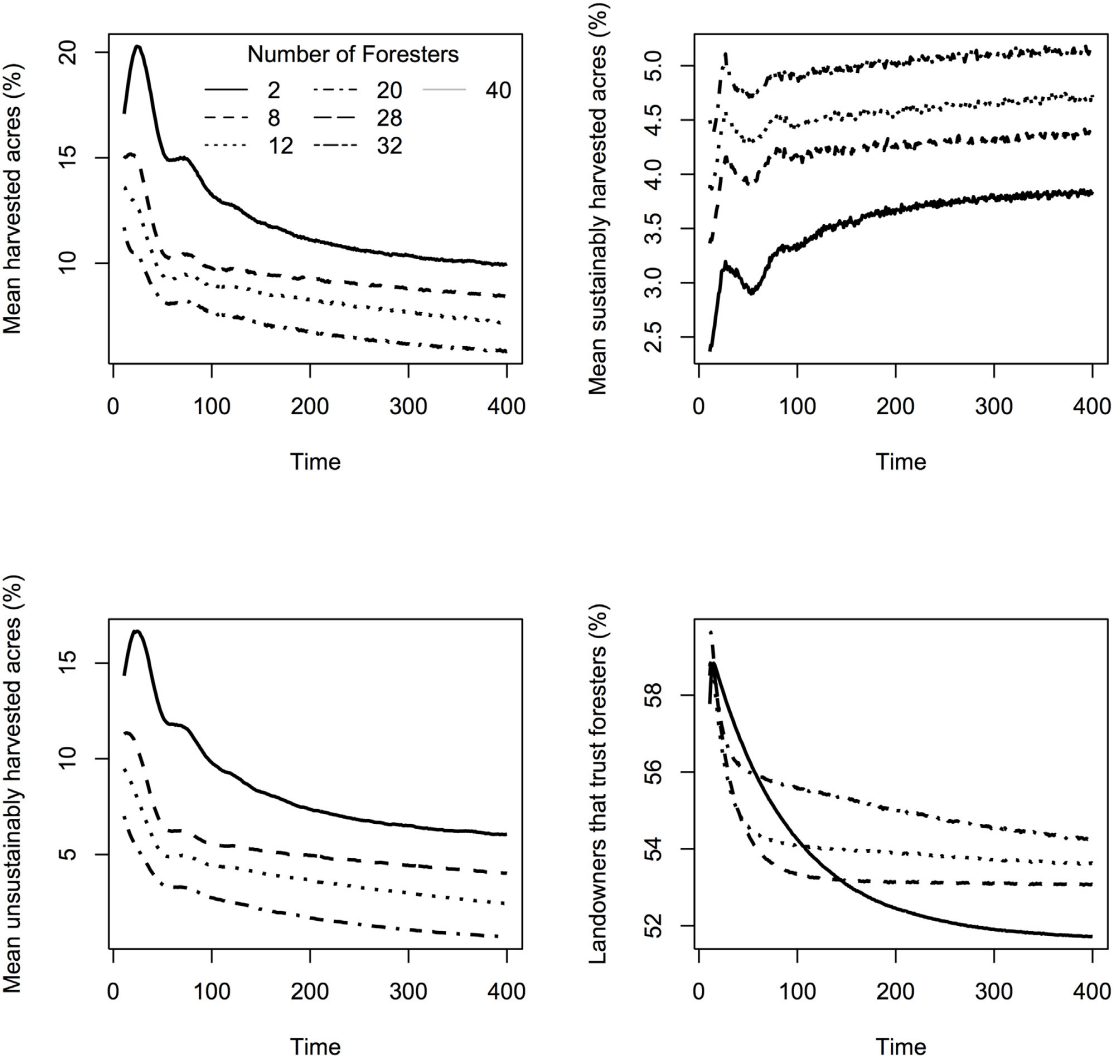
Percentage of the forest harvested and landowner trust in foresters over time for 4100 repetitions with seven different levels of foresters and 2 peer leaders.

To better describe the relationship between number of foresters/peer leaders on the landscape and trust or harvest level, we compared beanplots of the different levels. Beanplots are an alternative to boxplots for visual comparison of groups because they show a more detailed distribution and average of the group [46]. As foresters increased on the landscape, level of trust in foresters also increased, with the exception of 0–8 foresters (Fig 2). An examination of pairwise comparisons between number of peer leaders and number of foresters indicated significant differences between most levels (S1 Table). As foresters increased on the landscape, the percentage of forestland harvested decreased (Fig 2) while the percentage of that harvested forestland that was sustainably harvest increased (Fig 2). When number of peer leaders was held constant at 2, the more foresters on the landscape, the higher the amount of forest harvested sustainably compared to unsustainably (Fig 3). At all forester levels, trust increased initially, and then decreased. With a small number of foresters, trust decreased more rapidly over time.

**Fig 4:**
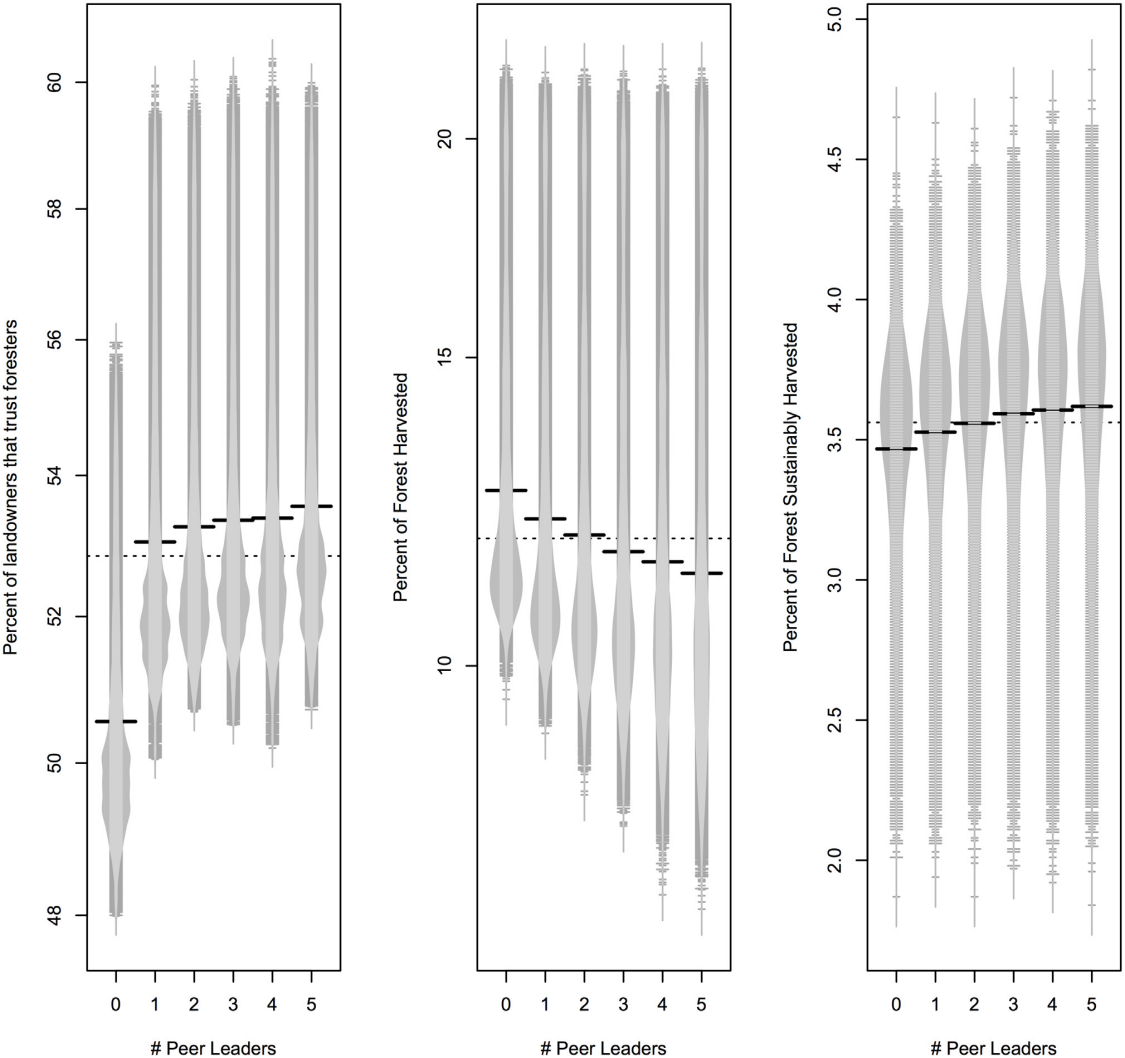
Beanplots [46] showing the distribution of level of landowner trust in foresters, percent of the forest harvested, and percent of the forest sustainably by the number of peer leaders. All other parameters are held constant and averaged for the last time step over 100 simulations per forester level. The dashed line is the overall mean of all peer leader levels.

**Fig 5:**
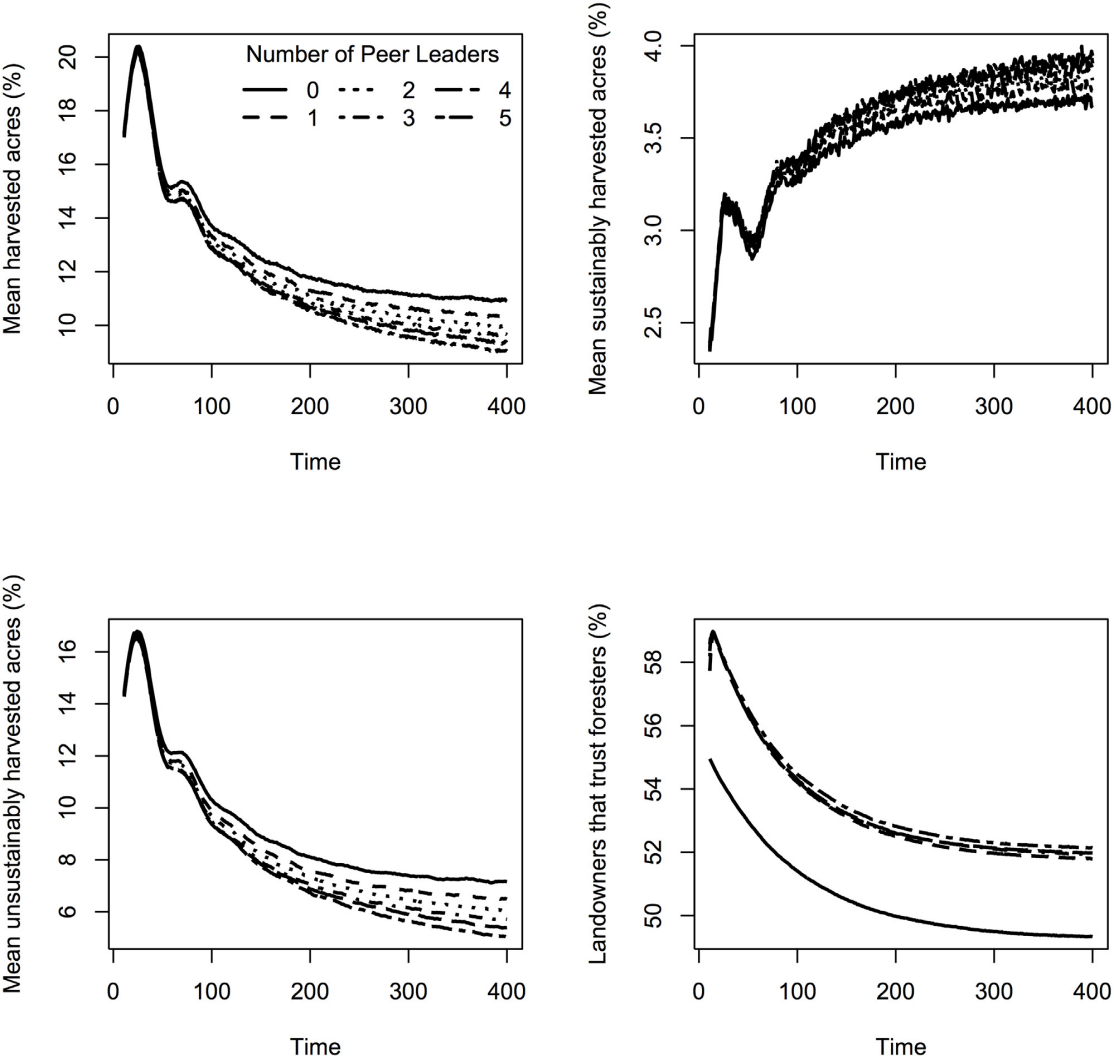
Harvest percentages and trust in foresters over time averaged for 100 repetitions with 6 different levels of peer leaders, a positive opinion type, and forester held constant at 20.

Although the percentage of the forest harvested and trust levels had some statistical difference by peer leader levels (S1 Table), there was no clear relationship as the number of peer leaders increased. For percentage of the forest harvested, there was no difference between 2–4 peer leaders, but some variation between 0 or 1 and 5 peer leaders (Fig 4). Percent of the forest that was sustainably harvested did not differ much by number of peer leaders on the landscape (Fig 4). Landowner trust in foresters was statistically lower when there were 0 peer leaders on the landscape compared to 1, 2, 3, 4, and 5 peer leaders (S1 Table, Fig 4), however there were no statistical differences between 2 and 1, 2 and 3, or 4 and 5 peer leaders on the landscape. As with the number of foresters on the landscape, when number of foresters was held constant, all levels of peer leaders led to a decrease in trust and decrease in percentage harvested over time (Fig 5).

Output from the boosted regression tree indicates that years/timestep and number of foresters had the most relative influence on model output (52.8% and 45.2%, respectively). Although trust in foresters was significantly different with different opinion types (Table 4), with positive opinions leading to higher trust than negative or neutral, opinion type was not an important influence on model output.

### Model validation

Construct and content validity was checked by comparing model constructs with the literature, subject matter experts, and commonly accepted definitions therein (S2 Table). We found that although the model is a reasonable imitation of the real-world and contains all the constructs intended in the model, there are additional constructs that will help the model imitate a real-world landscape of PWOs, further described in the Discussion section. Criterion, concurrent, and convergent validation checks were performed against similar models and available empirical data. We found that the percent of forest harvested was higher than harvest percentages reported in Massachusetts and Maine [15]. On average, PWOs harvest between 0.01–1.48% of the forested landscape annually, however these values are derived from landscapes that contain a mix of ownership types. As our landscape was entirely PWOs, the annual percentage is likely to be higher. Our model output indicated 5–21% of the landscape is harvested in a given year, but since PWOs comprise 33% of the ownership base in Maine, for example, our harvest percentages are closer to the national annual harvest percentages. Our logistic regression in the pre-interaction harvest decision sub-model was consistent with methods and output found in reviews of PWO timber harvesting behavior in the economics literature [26, 27].

## Discussion

Our results demonstrate the capability of this model to integrate social and ecological variables into a theoretic landscape of PWOs to predict sustainable timber harvesting and to explore the influence of landowner-forester and landowner-peer interactions on forest management decisions. We manipulated number of foresters, number of peer leaders, the type of opinion spread between landowners, and the number of landowner foresters and peer leaders could visit in a year. The number of foresters, number of peer leaders, and opinion type were statistically different regarding percentage of the landscape harvested and percentage of landowners that trust foresters. Similar ABMs include FLAME [15], ForestSim [13], and a model of spatial interaction and information flow [12]. Our model configuration and outputs are consistent with these models.

First, it was assumed that an interaction with a forester could lead to either increased or decreased trust in foresters, but that an interaction with a peer leader (someone trained in conservation who believes in the value of a forester’s advice) would lead to increased trust in foresters. Indeed, as the number of foresters increased on the landscape, so too did the level of trust in foresters. However, with zero foresters on the landscape, trust is high, indicating that although interactions between landowners and foresters can lead to an increase in trust, the stochasticity in whether trust increases or decreases brings the value of trust down compared to the stochasticity of initializing trust values and the increase in trust caused by peer leaders. These results suggest that having more foresters on the landscape will help build overall trust between landowners and foresters, but only if potential for loss of trust is carefully mitigated. The range between 16–24 foresters contained many non-significant pairwise comparisons, indicating that there is little difference in the forester-to-landowner ratio in the middle of the distribution, but larger effects if there are fewer or more foresters.

Second, it was assumed that harvesting would only take place if a landowner had made a decision to harvest, this decision was not changed by interaction with a forester or peer leader, and the harvestability of the parcel matched the landowner’s sustainability value. Percent harvested decreased over time with an initial wave of harvesting as mature forest was quickly converted to immature forest and landowner sustainability prevented many of the patches from being harvested when it was not advisable to do so. Sustainable harvesting typically increased slightly after the initial wave of cutting while unsustainable harvesting continued to decline, when there were foresters and peer leaders on the landscape influencing landowner sustainability.

Overall, the percentage of the landscape that was harvested decreased over time without positive opinion spread on the landscape. This is likely due to the eventual lack of harvestable parcels coupled with a regrowth submodel that doesn’t regrow enough forest in the 80 year period modeled. It is likely that if the model were run at least 100 years longer, trust and percent harvested would reach a point of equilibrium or oscillation, determined by the regrowth submodel.

Initial model output suggests that information flow between landowners and peer leaders and foresters are more likely to increase overall trust and lead to more sustainable harvesting choices. Support for peer-to-peer networking [8, 30] also suggests the critical role peer leaders may fill in bridging gaps that foresters cannot fill, whether because of their relationships (typically strangers) with landowners and their inability to work with enough landowners for 100% coverage. There is little research on PWO trust in information and natural resource professionals, although results from surveys suggest that only a small percentage of PWOs interact with these professionals on forest management issues [1] suggesting that this model will only be applicable to landscapes where natural resource professionals and peer leaders can successfully connect with the number of landowners represented in the model.

The model also suggests that with increased interactions and trust between landowners and foresters, PWOs are more likely to sustainably harvest their timber. Although the exact nature of the timber harvest is not explicitly modeled, there is evidence that landowners who are actively engaged (e.g., work with a forester, have a management plan, or belong to a woodland owner organization) tend to harvest more and are more willing to cooperate with one another regarding management activities [47]. Furthermore, Gootee et al. [29] found that landowners without a forestry background preferred information delivery in an empathetic and respectful manner as opposed to the typical expert vs. non-expert hierarchical manner, suggesting trust plays a role but so too does preference or learning style.

Finally, the model framework incorporates an opinion-spread function, whereby landowners pass information to either direct neighbors or random landowners on the landscape. Initial testing demonstrated that a positive opinion leads to increased trust across the landscape as compared to a neutral or negative opinion. This function is called an opinion, but could be interpreted more broadly as information sharing. Rosnow [48] summarizes the conditions likely to give rise to opinion: uncertainty, anxiety, outcome-related involvement, and credulity. Wang et al. [49] demonstrate that uncertainty can increase the speed at which opinions or gossip spread on a network, namely that rumors result from individuals making strategic choices rather than randomly sharing information. Information sharing can take place without these motivators and relates more to the relationships between people than individual motivation (e.g., people in permanent relationships tend to share accurate information since they are more accountable) [50]. The actual topology of information networks (e.g., specific links or relationships between landowners) has a demonstrated effect on natural resource management behaviors [51], so further elucidation of the relationships between landowners will be an important model improvement.

The proof of concept demonstrates that with a positive opinion spread on the landscape, sustainable forest harvesting increases and trust is maintained in over half of the landscape. Additionally, this proof of concept shows that landowner sustainability scores, general parcel characteristics, and landowner demographics can be tracked over time and model-wide averages can be calculated. After 80 years, landowners had aged significantly compared to the average age of 56.6 years with which the model was initialized. Sustainability scores were predominantly ‘1’ indicating that positive opinion spread may also impact the overall sustainability of landowners, providing more support for peer-to-peer networking.

This model could be used in many other contexts. It could be used to determine how information flow and trust impact individual decisions in urban contexts, using community activists as the “Influencers.” It could be used for other natural resource management issues, such as the opinion spread and water use decisions in areas with water shortages and water rights policy. Finally, the opinion spread submodel, which itself was modified from an existing NetLogo model, could be used in social science research whenever a rumor or opinion spread function may influence the decision or behavioral outcome of an individual. In these efforts, the modeler must identify the decision-making agent, the professionals providing expertise, and the influential peers that may change interactions between the agent and professional.

### Future model extensions

The goal of predictive modeling is to make the model as simple as possible, yet still capture relationships and variables necessary to answer foundational research questions. This model has several limitations that suggest possible extensions and additions, some of which are described below and most of which will require additional empirical data to correctly integrate them into this ABM framework. First, the relationship between landowner trust in foresters and peer leaders may require additional data on the relationship between trust and acceptance of information spread information. Future research should add a parameter that moderates the effect of interaction on landowners such as susceptibility to information or existing knowledge in addition to the trust parameter already in the model. Second, the amount of time that elapses between interactions should be recorded for each agent and moderate the extent to which interactions influence behavior. Future research could add explicit social network data such as a network topology to the model or empirical interaction patterns [52–55], add a utility or objective function that determines whether or not a harvest maximizes their ownership objectives allows PWOs to thin or partially cut a stand, and test other decision making models besides logistic regression in the pre-interaction harvest decision. Finally, it is highly recommended that future research quantifies the effect of psychological distance on PWOs and uses the model as an experimental framework for testing the effect of it on forest management decisions.

Although several scholars and practitioners were solicited for advice on model parameterization, stakeholder knowledge could be more rigorously incorporated into model construction and improvement [56].

## Conclusion

This theoretic model of PWO timber harvesting behavior and interaction will increase understanding of the social and ecological factors underlying forest management behaviors and inform hypotheses for future empirical research. In particular, modeling efforts like this provide an important testing ground for new theories, relationships between variables, and deeper understanding of feedbacks in social-ecological systems. This model can help landscape-scale natural resource managers allocate resources to efficiently provide outreach to local forested areas. By focusing on trust building instead of increasing the number of professionals available to landowners, sustainable forest management may be increased. Furthermore, by understanding landowner communication networks, peer leaders can be trained as liaisons between forestry professionals and landowners, increasing the effectiveness of limited personnel and budgetary constraints. Finally, decades of research on PWO timber harvesting behavior can be included in a modeling framework that allows researchers to understand not only internal cognitive factors and measureable demographics, but also external drivers such as social interactions and information flow.

## Supporting Information

S1 Table Agent-based model verification. Pairwise comparisons of number of foresters on the landscape.
(PDF)

S2 Table Agent-based model validation. Definitions and constructs used in this agent-based model
(PDF)
