# Supplementary material for: An Agent-Based Model of Private Woodland Owner Management Behavior Using Social Interactions, Information Flow, and Peer-To-Peer Networks
Source: PLoS One. 2015 Nov 12;10(11):e0142453. doi: 10.1371/journal.pone.0142453 (PMC4642987; doi:10.1371/journal.pone.0142453)
Supplement: S1 Table — Pairwise comparisons of number of foresters on the landscape. (PDF) [file pone.0142453.s001.pdf]

S1 Table: Agent-based model verification

Pairwise comparisons of number of foresters on the landscape. Significance (x) was determined at the 0.05 level, compared to non-significance (ns). The top or right half of the matrix is based on percentage of the landscape harvested while the bottom or left side of the matrix is based on landowner trust in foresters.

Foresters	0	2	4	6	8	10	12	14	16	18	20	22	24	26	28	30	32	34	36	38	40
0		x	x	x	x	x	x	x	x	x	x	x	x	x	x	x	x	x	x	x	x
2	x		x	x	x	x	x	x	x	x	x	x	x	x	x	x	x	x	x	x	x
4	x	x		x	x	x	x	x	x	x	x	x	x	x	x	x	x	x	x	x	x
6	x	x	x		x	x	x	x	x	x	x	x	x	x	x	x	x	x	x	x	x
8	x	x	x	ns		x	x	x	x	x	x	x	x	x	x	x	x	x	x	x	x
10	x	x	x	x	x		ns	x	x	x	x	x	x	x	x	x	x	x	x	x	x
12	x	x	x	x	x	x		x	x	x	x	x	x	x	x	x	x	x	x	x	x
14	x	x	x	x	x	x	x		x	x	x	x	x	x	x	x	x	x	x	x	x
16	x	x	x	x	x	x	x	x		ns	x	x	x	x	x	x	x	x	x	x	x
18	x	x	x	x	x	x	x	x	x		x	x	x	x	x	x	x	x	x	x	x
20	x	x	x	x	x	x	x	x	x	x		ns	x	x	x	x	x	x	x	x	x
22	x	x	x	x	x	x	x	x	x	x	ns		ns	x	x	x	x	x	x	x	x
24	x	x	x	x	x	x	x	x	x	x	x	x		ns	x	x	x	x	x	x	x
26	x	x	x	x	x	x	x	x	x	x	x	x	x		ns	ns	x	x	x	x	x
28	x	x	x	x	x	x	x	x	x	x	x	x	x	x		ns	x	x	x	x	x
30	x	x	x	x	x	x	x	x	x	x	x	x	x	x	x		ns	x	x	x	x
32	x	x	x	x	x	x	x	x	x	x	x	x	x	x	x	x		ns	ns	ns	x
34	x	x	x	x	x	x	x	x	x	x	x	x	x	x	x	x	x		ns	ns	ns
36	x	x	x	x	x	x	x	x	x	x	x	x	x	x	x	x	x	x		ns	ns
38	x	x	x	x	x	x	x	x	x	x	x	x	x	x	x	x	x	x	x		ns
40	x	x	x	x	x	x	x	x	x	x	x	x	x	x	x	x	x	x	x	ns	

Pairwise comparisons of number of peer leaders on the landscape. Significance (x) was determined at the 0.05 level, compared to non-significance (ns). The top or right half of the matrix is based on percentage of the landscape harvested while the bottom or left side of the matrix is based on landowner trust in foresters.

Peer Leaders	0	1	2	3	4	5
0		ns	x	x	x	x
1	x		ns	ns	ns	x
2	x	ns		x	ns	ns
3	x	x	ns		ns	ns
4	x	x	x	x		ns
5	x	x	x	x	ns	
