# Supplementary material for: An Agent-Based Model of Private Woodland Owner Management Behavior Using Social Interactions, Information Flow, and Peer-To-Peer Networks
Source: PLoS One. 2015 Nov 12;10(11):e0142453. doi: 10.1371/journal.pone.0142453 (PMC4642987; doi:10.1371/journal.pone.0142453)
Supplement: S2 Table — Definitions and constructs used in this agent-based model (PDF) [file pone.0142453.s002.pdf]

S2 Table: Agent-based model validation

Definitions and constructs used in this agent-based model

Term	Definition	Model Parameter	Source(s)
Ownership Objectives	Reason for owning woodland	Yes	Butler, Leatherberry, & Williams 2005
Sustainable harvesting	Removal of merchantable timber such that forest regeneration occurs and desirable species and growth rates are maintained	Landowner sustainability inclination, Record of sustainable harvest (no record of volume removed)	Peterken, 1981; Hunter, 1990, Kohm & Franklin, 1997
Forester	A professional trained in forestry with a B.S. or M.F in forestry, who is licensed and certified	Yes	Society of American Foresters, Dictionary of Forestry
Peer Leader	An individual trained in conservation that is responsible for some type of conservation role in a town or community	Yes	Keystone Cooperator Program, Peer-to-peer networking literature
Psychological distance	An individual's mental representations of objects and activities that depend on various dimensions of distance between the individual and the object	Yes	Trope & Liberman, 2003, see Chapter 3
Trust	"an expectancy held by an individual or group that word, promise, verbal, or written statement of another individual or group can be relied on"	Yes	Rotter, 1971
Timber harvesting	The decision to harvest	Decision is a parameter, actual	See Chapter 1

behavior	timber commercially (beyond personal use) as measured by an actual harvest taking place	harvest is an output.	
